# Cardiac Adverse Reactions With COVID-19 Vaccinations

**DOI:** 10.7759/cureus.21372

**Published:** 2022-01-18

**Authors:** Mikhail L De Jesus, Jevin Yabut, Manish Kumar, Joyce Meng

**Affiliations:** 1 Medicine, University of Connecticut Health, Farmington, USA; 2 Cardiology, University of Connecticut Health, Farmington, USA

**Keywords:** vaccine adverse reaction, pericarditis, myocarditis, vaccine, covid-19

## Abstract

The coronavirus disease 2019 (COVID-19) pandemic has resulted in millions of deaths worldwide. The emergency use authorization for both the Pfizer-BioNTech and Moderna mRNA COVID-19 vaccinations was a major turning point in the battle against COVID-19. These vaccines have been well-tolerated; however, there have been reported cases of myocarditis and pericarditis after receiving the second dose of the vaccine. We present two cases of myocarditis and pericarditis that occurred after receiving the COVID-19 vaccination. Although there are other potential etiologies that could explain myocarditis and pericarditis in these cases, it is important to consider the COVID-19 vaccine as a plausible cause. More research is required to investigate the potential adverse effects of the available COVID-19 vaccines.

## Introduction

Since the start of the severe acute respiratory syndrome coronavirus 2 (SARS-CoV-2) (coronavirus disease 2019 (COVID-19)) pandemic, there have been over 4.5 million deaths worldwide [1]. The emergency use authorization for both the Pfizer-BioNTech and Moderna mRNA COVID-19 vaccinations in December 2020 was a major turning point in the battle against COVID-19. These vaccines largely have been well-tolerated; however, there are multiple cases of myopericarditis reported in association with vaccination [2-6]. We present two cases of pericarditis or myocarditis suspected to be secondary to COVID-19 mRNA vaccination.

## Case presentation

Case 1

**Figure 1:**
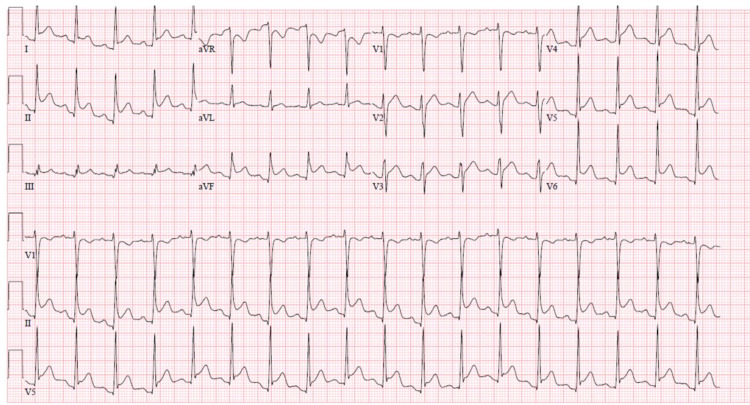
ECG from Case 1 showing diffuse ST-segment elevation, downsloping TP segment, and PR depression.

A healthy 28-year-old male presented to the hospital with midsternal chest pain that started suddenly, radiated to the left arm, worsened with deep inspiration and lying flat, and improved with sitting upright. The patient reported associated headaches, fever, hemoptysis, and unilateral leg swelling. On presentation, he had a temperature of 100.2°F, tachycardia of 101 beats per minute, blood pressure of 148/74 mmHg, and oxygen saturation of 95% on room air. Physical exam was remarkable for a hyperdynamic precordium with loud S1 and no significant murmur. Electrocardiogram (ECG) revealed diffuse ST-segment elevation in multiple leads, downsloping TP segment, and PR segment depression in lead II (Figure 1). Laboratory work showed an elevated white cell count of 15.7 10*3/µL with 79.1% neutrophils and normal troponin I level measuring 0.01 ng/mL. Chest radiograph showed no acute cardiopulmonary process. Transthoracic echocardiogram (TTE) showed normal left ventricular ejection fraction (LVEF) of 55-65%, no regional wall motion abnormality, and no pericardial effusion. Based on his characteristic chest pain and ECG changes, the diagnosis of acute pericarditis was made. He was started on colchicine and ibuprofen. Notably, he had received the second dose of the Moderna COVID-19 vaccine 10 days prior to presentation. Given the absence of obvious etiology, it was thought that his presentation might have been due to an adverse effect of the vaccine. His symptoms as well as ECG changes resolved within one month.

Case 2

**Figure 2:**
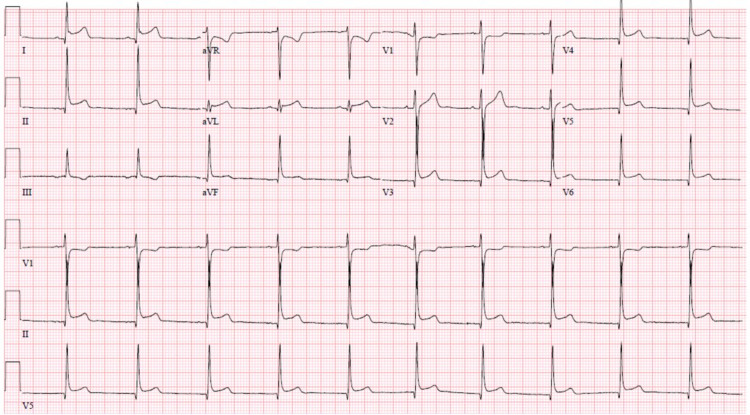
ECG from Case 2 showing diffuse ST-segment elevation.

An 18-year-old male presented with subjective fevers, myalgias, and substernal chest pain radiating down the left arm. The pain was noted to improve when standing and was unrelated to exertional activity. His vital signs and physical examination were unremarkable. SARS-COV-2 polymerase chain reaction and influenza A and B antigen testing were negative. Laboratory investigations were significant for an elevated C-reactive protein of 23.1 mg/L, elevated sedimentation rate of 20 mm, and elevated troponin I of 6.4 ng/mL. Repeat troponin I subsequently peaked at 11.74 ng/mL. ECG revealed normal sinus rhythm with diffuse ST-segment elevations except in leads V1 and aVR (Figure 2). The chest radiograph was unremarkable. TTE revealed a low normal LVEF of 50-55% with no regional wall abnormalities. He received the second dose of the Pfizer-BioNTech COVID-19 vaccine five days prior to the onset of his symptoms. He was diagnosed with suspected COVID-19 vaccine-related myopericarditis given his complaint of positional chest pain, ECG changes consistent with pericarditis, and elevated troponin I. He was prescribed colchicine and lisinopril. Repeat TTE one month later revealed no change in his LVEF.

## Discussion

As of August 2021, there have been over 4.5 million deaths worldwide from SARS-CoV-2 infection [1]. To date, there have been over 5 million vaccines administered worldwide after the emergency use authorization for both the Pfizer-BioNTech and Moderna mRNA COVID-19 vaccinations in December 2020 [1]. The vaccines have been largely well-tolerated with mostly self-limiting adverse effects such as pain, swelling, redness at the site of injection, and systemic symptoms such as fever [2]. More serious side effects, including myocarditis, have been reported after receiving the COVID-19 mRNA vaccine predominantly in young males of 16-18 years of age [2-6]. The majority of the cases associated with COVID-19 vaccinations are reported after the second dose with the onset of symptoms at a median of three days after vaccination [3]. Approximately 86% of the patients present with chest pain, 61% with ST- or T-wave changes on EKG, 64% of patients with elevations in cardiac enzymes, and 17% having abnormal cardiac imaging [3].

We report two separate cases of myopericarditis that occurred within a few days to weeks of COVID-19 vaccination. Our patients presented with commonly reported symptoms and clinical findings consistent with myopericarditis. The onset of their symptoms was shortly after receiving a dose of the COVID-19 mRNA vaccination, consistent with the timelines typically reported by the Vaccine Adverse Event Reporting System (VAERS). Almost all patients returned to baseline within a few weeks.

The proposed mechanism of myopericarditis caused by COVID-19 mRNA vaccines involves the body’s innate immune response to RNA [3]. RNA is known to be immunogenic and had precluded mRNA vaccines in the past [3]. Scientists have learned to modify nucleosides to suppress immunogenicity allowing for the development of mRNA-based vaccines [7]. It is proposed that some persons have a genetic predisposition that results in the aberrant activation of the innate and acquired immune response [3]. In these individuals, it is proposed that the mRNA vaccine is detected as an antigen by the immune system leading to inflammation and immunologic activation [3].

## Conclusions

The patients in our case series did not have other obvious etiologies for their myocarditis or pericarditis. The most common etiology of pericarditis is idiopathic/viral, and often, symptoms of antecedent viral infection may not be ascertained in many patients. It is possible that these two patients may have had symptoms of viral infection that were not significant enough in the preceding days to weeks prior to the presentation. Despite these confounding factors, these two patients presented within days to weeks after vaccination, which raises the suspicion that the etiology of myocarditis or pericarditis may have been an adverse effect of vaccination. It cannot be said with certainty if their presentation within days to weeks of vaccination was a coincidence or merely an association. Large-scale data are currently being collected regarding cardiac adverse effects of vaccination. Clinicians should be aware of the possible association between the COVID-19 vaccine and myocarditis and pericarditis. It should be emphasized that this rare adverse effect should not preclude clinicians from prescribing the vaccine given its favorable risks and benefits.
